# Availability and utilization of malaria prevention strategies in pregnancy in eastern India

**DOI:** 10.1186/1471-2458-10-557

**Published:** 2010-09-17

**Authors:** Blair J Wylie, Ahmar H Hashmi, Neeru Singh, Mrigendra P Singh, Jordan Tuchman, Mobassir Hussain, Lora Sabin, Kojo Yeboah-Antwi, Camellia Banerjee, Mohamad I Brooks, Meghna Desai, Venkatachalam Udhayakumar, William B MacLeod, Aditya P Dash, Davidson H Hamer

**Affiliations:** 1 Center for Global Health and Development, Boston University School of Public Health, Boston, MA, USA; 2 Department of International Health, Boston University School of Public Health, Boston, MA, USA; 3 Division of Maternal-Fetal Medicine, Department of Obstetrics and Gynecology, Massachusetts General Hospital and Harvard Medical School, Boston, MA, USA; 4 National Institute of Malaria Research Field Station, Jabalpur, India; 5 Regional Medical Research Centre for Tribals (Indian Council for Medical Research), Jabalpur, India; 6 Center for Leadership and Management, Management Sciences for Health, Cambridge, MA, USA; 7 Malaria Branch, Division of Parasitic Diseases, National Center for Zoonotic Vector Borne and Enteric Diseases, Centers for Disease Control and Prevention, Atlanta, GA, USA; 8 National Institute of Malaria Research, Delhi, India; 9 Section of Infectious Diseases, Department of Medicine, Boston University School of Medicine, Boston, MA, USA

## Abstract

**Background:**

Malaria in pregnancy in India, as elsewhere, is responsible for maternal anemia and adverse pregnancy outcomes such as low birth weight and preterm birth.

It is not known whether prevention and treatment strategies for malaria in pregnancy (case management, insecticide-treated bednets, intermittent preventive therapy) are widely utilized in India.

**Methods:**

This cross-sectional study was conducted during 2006-2008 in two states of India, Jharkhand and Chhattisgarh, at 7 facilities representing a range of rural and urban populations and areas of more versus less stable malaria transmission. 280 antenatal visits (40/site) were observed by study personnel coupled with exit interviews of pregnant women to assess emphasis upon, availability and utilization of malaria prevention practices by health workers and pregnant women. The facilities were assessed for the availability of antimalarials, lab supplies and bednets.

**Results:**

All participating facilities were equipped to perform malaria blood smears; none used rapid diagnostic tests. Chloroquine, endorsed for chemoprophylaxis during pregnancy by the government at the time of the study, was stocked regularly at all facilities although the quantity stocked varied. Availability of alternative antimalarials for use in pregnancy was less consistent. In Jharkhand, no health worker recommended bednet use during the antenatal visit yet over 90% of pregnant women had bednets in their household. In Chhattisgarh, bednets were available at all facilities but only 14.4% of health workers recommended their use. 40% of the pregnant women interviewed had bednets in their household. Only 1.4% of all households owned an insecticide-treated bednet; yet 40% of all women reported their households had been sprayed with insecticide. Antimalarial chemoprophylaxis with chloroquine was prescribed in only 2 (0.7%) and intermittent preventive therapy prescribed in only one (0.4%) of the 280 observed visits.

**Conclusions:**

A disconnect remains between routine antenatal practices in India and known strategies to prevent and treat malaria in pregnancy. Prevention strategies, in particular the use of insecticide-treated bednets, are underutilized. Gaps highlighted by this study combined with recent estimates of the prevalence of malaria during pregnancy in these areas should be used to revise governmental policy and target increased educational efforts among health care workers and pregnant women.

## Background

Infection with malaria during pregnancy carries significant risks to both mother and baby. An estimated 50 million pregnancies worldwide occur in areas where malaria is a concern [1]. In areas of low or unstable malaria transmission, pregnant women have little acquired immunity to malaria and are at increased risk of symptomatic malaria, severe malaria, anemia and adverse birth outcomes such as miscarriage, preterm labor or stillbirth [2,3]. In areas of stable transmission, symptomatic maternal infections occur less frequently, yet the mother remains at risk for severe anemia and the fetus at risk for low birth weight and consequently perinatal death secondary to impaired growth and/or preterm birth [1,2,4,5].

Ninety-five percent of India's population is at risk for malaria with an estimated 2 to 3 million cases reported annually nationwide [6,7]. Malaria transmission in India is predominantly unstable with pockets of more stable transmission [6]. Although less studied than exposed pregnancies in sub-Saharan Africa, epidemiological studies of malaria in pregnancy in India have demonstrated similar adverse pregnancy outcomes such as anemia, preterm labor, stillbirth and low birth weight [8-12].

Strategies for prevention and control of malaria during pregnancy include prompt recognition and treatment of women with symptomatic illness (case management) and a package of prevention measures encompassing use of insecticide-treated bednets (ITNs) and provision of intermittent preventive therapy in pregnancy (IPTp) [13]. In India, the government has initiated measures to improve malaria prevention and treatment, with a special emphasis on pregnant women and children. Several states have benefitted from the Enhanced Malaria Control Project funded by the World Bank from 1997 to 2005, which emphasized an integrated approach to malaria control including early case detection and prompt treatment, vector control with targeted indoor residual spraying, distribution of ITNs, increased public awareness through informational campaigns and strengthening of regional institutions to provide malaria surveillance and control of outbreaks [14,15]. In addition, the official policy endorsed by the National Vector Borne Disease Control Program through 2007 recommended chloroquine chemoprophylaxis during pregnancy for women living in high-risk areas [16].

It is not known whether this comprehensive pregnancy malaria control strategy that exists on paper has been widely adopted in India. We conducted this study to assess the knowledge, availability and utilization of malaria prevention and treatment strategies among pregnant women and health care workers (HCWs) in two malaria-endemic states of eastern India in order to inform policy recommendations.

## Methods

### Study Sites

This study was one component of two larger cross-sectional surveys conducted in the states of Jharkhand and Chhattisgarh that assessed the burden of malaria in pregnancy using rapid field assessment methodology developed by the United States Centers for Disease Control and Prevention [17]. Jharkhand and Chhattisgarh were chosen because they contribute 7% and 13%, respectively, of all annually reported malaria cases in India [6]. Seven antenatal clinics (ANCs), three in Jharkhand and four in Chhattisgarh, were purposively selected in order to include a range of rural and urban populations and areas of more versus less stable malaria transmission.

**Figure 1:**
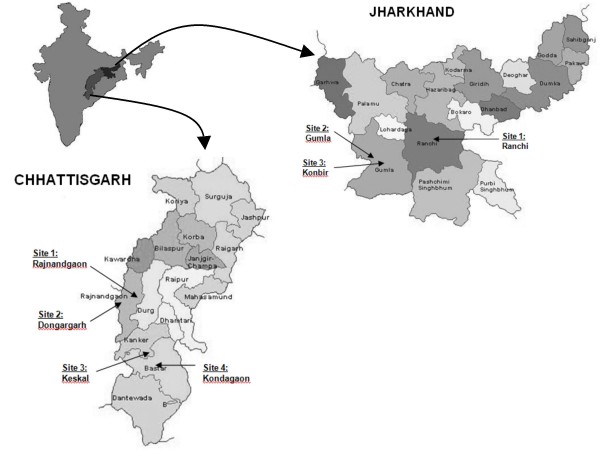
**Map of Jharkhand and Chhattisgarh states with study sites identified**. Arrows indicate the health facilities where data collection took place: Jharkhand state (*Ranchi*- urban; *Gumla*- semiurban; *Konbir*- rural) and Chhattisgarh state (Bastar district, stable transmission: *Keskal*- rural and *Kondagaon*- urban; Rajnandgaon district, unstable transmission: *Rajnandgaon*- urban and *Dongargarh*- rural).

In Jharkhand (Figure 1), we chose three facilities to ensure inclusion of rural, semi-urban and urban populations. The Ursula Mission Hospital serves a predominantly rural population and is situated in Konbir (Gumla district). Approximately 1200 ANC visits are performed at this hospital each year. Konbir is situated very close to the border of Simdega, a highly malarious district with a slide positive rate (SPR) of 14% in 2005 [18]. Civil Hospital, the district hospital for Gumla district, serves a semi-urban population and performs 2000 ANC visits annually. The SPR for Gumla district was 3.4% in 2005 with substantially higher rates reported in the recent past, ranging from 10% to 19.7% between 1997 and 2003 [18]. Sadar Hospital, the district hospital in Ranchi, is located in an urban location averaging between 3200 and 3600 annual ANC visits. The SPR for Ranchi district was 7.2% in 2005 [18].

The study protocol for Chhattisgarh was designed after initiation of research activities in Jharkhand with a slightly different aim of enrolling women in areas of more stable versus less stable malaria transmission rather than based on urbanity alone. In Chhattisgarh (Figure 1), two districts were targeted. Bastar district has stable, seasonal transmission of malaria while Rajnandgaon district is characterized by unstable transmission. The SPR in Bastar district was 12.1% in 2005 [19]. In Rajnandgaon, the SPR was 1.6% in 2005. Within each district, we selected both a rural and an urban ANC. Rural antenatal facilities chosen were the Keskal Community Health Center (Bastar district) and Dongargarh Community Health Center (Rajnandgaon district) with approximately 750 and 1500 ANC visits per year, respectively. The urban ANCs were located at Kondagaon Ravindra Nath Taigore Community Health Center (Bastar district) and at District Hospital Rajnandgaon (Rajnandgaon district) and reported an average of 1500 and 2450 ANC visits per year, respectively.

Data from our contemporaneous cross-sectional surveys of unselected pregnant women presenting for ANC care during a twelve month period at these same study sites revealed a prevalence of peripheral parasitemia of 1.8% in Jharkhand [20] and 1.2% in Chhattisgarh (2.8% in Bastar district and 0% in Rajnandgaon district) [21]. The prevalence of placental parasitemia among women presenting for delivery at these study sites during the same time period was 2.4% in Jharkhand and 3.1% in Chhattisgarh (3.6% in Bastar district and 3.2% in Rajnandgaon district) [20,22].

### Paired ANC observations and exit interviews

Pregnant women presenting for antenatal care at the participating ANC facilities were approached for study participation. Research nurses were stationed at the participating antenatal clinics and gave a brief overview of the study to pregnant women waiting to be seen for antenatal care. Women interested in participation were enrolled if they were ≥15 years of age, had not previously participated in the study and were willing to provide informed consent. Trained study personnel observed the antenatal visit conducted by the HCW, noting, with the aid of a checklist, what history was taken, parts of the physical exam were performed, laboratory tests were ordered and health information was communicated. The study nurse accompanied the research subject to the laboratory and the pharmacy to verify the laboratory tests ordered and the medications prescribed. Following the ANC visit, the study nurse conducted an exit interview with the subject. Information collected included socio-demographics, malaria prevention measures used during pregnancy and satisfaction with the health information received during the antenatal visit. Specific malaria prevention measures discussed included ownership of a bednet, treatment of the bednet with an insecticide, personal use of the bednet on the prior night and on most nights, use of antimalarial chemoprophylaxis, and household insecticide spraying by the government at any time point. Upon completion of a paired HCW observation and client exit interview, recruitment of additional subjects from those waiting to be seen for antenatal care was carried out until the targeted number of 40 per site was achieved. HCWs at each of the participating ANC sites were oriented to the study objectives and procedures prior to study initiation and agreed to have their antenatal visits observed. Observed ANC visits included both initial prenatal appointments as well as return visits.

### Facility assessments

Study personnel conducted a structured facility assessment survey at each of the seven participating ANCs. Along with background information about the facilities, study personnel focused on four key areas: antenatal services customarily provided, antenatal statistics routinely collected, malaria diagnostic capabilities and availability of bednets and antimalarial drugs. Interviewers consulted physicians, block medical officers, dispensary attendants and laboratory technicians to complete these assessments. In addition to detailed questions, an inventory of stocked supplies and drugs was performed. Inventory records were reviewed to identify relevant malaria supply stockouts in the previous six months.

### Ethical clearance

This study was approved by the Institutional Review Boards of Boston University and the Centers for Disease Control and Prevention, the Ethics Committee of the National Institute of Malaria Research in India, the Scientific Advisory Committee of the National Institute of Malaria Research and the Health Ministry Screening Committee of the Indian Council of Medical Research.

### Data management and analysis

For the paired ANC observations-exit interviews, case report forms and study procedures were evaluated in a pilot study and modified accordingly. All case report forms were checked for completeness and inappropriate or illogical responses. The forms were double-entered using CS-Pro Version 4.0 (United States Census Bureau, Washington, DC), with range, consistency and edit checks built into the data entry program for quality control. The two databases were validated and all inconsistencies and differences were resolved. Statistical analyses were performed using SAS software version 9.1 (Cary, North Carolina). Categorical data are presented as frequency counts (percent) and compared using the chi-square or Fisher's exact statistic as appropriate. Since most participants did not know their exact date of birth, we categorized subjects' ages in ranges based on their estimations.

## Results

### Paired ANC observations and exit interviews

Recruitment and enrollment took place during December 2006 through January 2007 in Jharkhand and December 2007 to March 2008 in Chhattisgarh. In Jharkhand, 120 pregnant women were enrolled, while 160 pregnant women were enrolled in Chhattisgarh (40 per ANC site). The proportion of ANC visits observed during the study period ranged from 5% to 20%, varying from site to site depending on the volume of total ANC visits at each site. Table 1 provides the socio-demographic characteristics of subjects enrolled in the ANC observations-exit interviews. Of all subjects, nearly all were married (99.6%) and most (77.5%) were engaged in household work. Hindi was the predominant language spoken at home (99.2%). Nearly one-fifth of subjects had never received formal schooling. The vast majority were members of traditionally disadvantaged populations now given administrative recognition: "Scheduled Tribes" (26.1%), originally indigenous people who are now fairly integrated with mainstream society; "Other Backward Castes" (33.9%), encompassing diverse groups not included in other classifications; and, "Scheduled Castes" (23.6%), primarily consisting of historically lower castes. 16.4% were "General Caste", comprised of all other individuals, including formerly higher castes.

**Table 1:** Enrolled Pregnant Women Demographics

	Jharkhand n = 120	Chhattisgarh n = 160
Age (years)		
15-19	9.2%	7.5%
20-34	85.0%	90.0%
> 35	5.8%	2.5%
Married	100%	99.4%
Hindi primary language spoken	100%	98.8%
Caste		
General Caste	12.5%	19.4%
Schedule Caste	26.7%	21.3%
Other Backward Caste	30.8%	36.3%
Schedule Tribe	30.0%	23.1%
No formal schooling*	20.8%	14.4%
Primary occupation		
Housework	78.3%	76.9%
Farming	7.5%	12.5%
Other	14.2%	10.6%

* p = 0.002 comparing Jharkhand with Chhattisgarh participants. No other significant differences between study sites with regard to participant demographics.

Table 2 details key observations made during the antenatal visits by research staff. Greater than 99% of the ANC visits were conducted by a physician. In general, an evaluation for anemia by history, physical examination and/or lab testing was more common than assessments for other signs or symptoms of malaria (history of fever, measurement of temperature, ordering blood smear). Hemoglobin levels were measured at nearly three-fourths of all visits (72.8%) whereas blood smears were obtained for about one-quarter of subjects (27.5%). Both hemoglobin and blood smears were ordered significantly more often by HCWs in Chhattisgarh than in Jharkhand. In Jharkhand, malaria was rarely discussed and bednets were never recommended. There was more of a focus on malaria in Chhattisgarh with one quarter of HCWs reviewing malaria protection measures with a specific mention of bednets in 15.6% of the discussions. In Chhattisgarh, HCWs were more likely to recommend a bednet in Bastar district, with stable malaria transmission compared with Rajnandgaon district, with unstable transmission (24% vs. 5%, p = 0.002). Antimalarial prophylaxis was rarely prescribed in either state. Chloroquine chemoprophylaxis was prescribed in only 2 (0.7%) and intermittent preventive therapy prescribed in only one (0.4%) of the 280 overall observed visits.

**Table 2:** Health Care Worker Observations

	Jharkhand n = 120	Chhattisgarh n = 160
Asks about presence of fever*	20.0%	48.1%
Measures temperature	17.5%	15.6%
Obtains blood smear*	14.2%	37.5%
Assesses for signs/symptoms of anemia*	40.8%	75.0%
Checks hemoglobin*	54.2%	86.9%
Discusses malaria protection measures*	0.8%	25.0%
Prescribes antimalarial prophylaxis	0.8%	1.3%
Recommends bednet use*	0%	15.6%
Level of HCW training		
Doctor	99.2%	100%
Nurse	0.8%	0%

HCW = health care worker.

* p < 0.001 comparing Jharkhand with Chhattisgarh participants.

The most widely utilized malaria prevention measure was sleeping under a bednet (Table 3). Over 90% of subjects in Jharkhand had a bednet in their household and the vast majority (82.5%) had slept under it the prior night. Household bednet availability was much lower in Chhattisgarh (40% of households) and only one-half of subjects with an available bednet had slept under it the prior night. In both states, most bednets were untreated, not ITNs. In contrast, 40% of all women reported that their households had been sprayed with insecticide by the government at some point. As anticipated from the observed ANC visits, use of antimalarial chemoprophylaxis during the pregnancy was extremely rare in both states with only one participant, who was unaware of the exact medicine prescribed, reporting use of chemoprophylaxis.

**Table 3:** Utilization of malaria prevention measures by pregnant women

	Jharkhand n = 120	Chhattisgarh n = 160
Reports malaria as a large concern in pregnancy*	76.7%	36.3%
Bednet available in household*	90.8%	40%
Slept under bednet last night*	82.5%	20%
Insecticide-treated bednet in household^†^	3.3%	0%
Use of chemoprophylaxis in pregnancy	0%	0.6%
Government ever sprayed house with insecticide^¶^	50.8%	31.9%

* p < 0.001 comparing Jharkhand with Chhattisgarh participants.

^† ^p = 0.02 comparing Jharkhand with Chhattisgarh participants.

^¶ ^p = 0.001 comparing Jharkhand with Chhattisgarh participants.

### Facility Assessments

Health center assessments surveys were conducted by study personnel at each of the 7 participating ANC facilities during the month of December 2006 in Jharkhand and November 2007 in Chhattisgarh. All facilities had the onsite capability to perform and diagnose malaria by blood smear, with the exception of the district hospital in Rajnandgaon, whose microscope was not functional at the time of evaluation (Table 4). Rapid diagnostic tests were not available at any of the surveyed facilities. Chloroquine was available at all facilities and was the most widely and well stocked antimalarial. The stock of chloroquine ranged in quantity from 500 tablets in Gumla (Jharkhand) to 15,000 tablets in Dongargarh (Chhattisgarh). Only 1 of the 7 facilities had experienced a chloroquine stockout, defined as a one month period of time without drug, in the six months preceding the assessment. Sulfadoxine-pyrimethamine, widely used in sub-Saharan Africa for IPTp, was available at only 3 of the 7 facilities and was absent from the largest ANC in each state (Ranchi and Rajnandgaon). Artesunate, utilized for the treatment of uncomplicated malaria in nonpregnant adults, usually as part of combination therapy with a second drug and increasingly used in pregnant women beyond the first trimester, was available at only 1 site. Among injectable antimalarials, arte-ether was available more commonly than quinine. Iron and folic acid supplements were provided to all women at each health center but one facility had none in stock at the time of the evaluation (Gumla).

**Table 4:** Facility Assessments

	Jharkhand			Chhattisgarh			
	Gumla (Semi-urban)	Konbir (Rural)	Ranchi (Urban)	Rajnandgaon (Urban)	Dongargarh (Rural)	Kondagaon (Urban)	Keskal (Rural)
Bednets available for PW	**√**			**√**	**√**	**√**	**√**
Insecticide-treated bednets	**√**			some	**√**		**√**
Average # bednets distrubuted/month	2200*			200	30	20	5
Antimalarials in stock							
Chloroquine	**√**	**√**	**√**	**√**	**√**	**√**	**√**
Quinine (injectable)	**√**	**√**					**√**
SP	**√**	**√**					
Artesunate	**√**						
Arteether (injectable)	**√**	**√**	**√**	**√**	**√**	**√**	**√**
Micronutrients in stock							
Multivitamin		**√**	**√**	**√**	**√**		**√**
Iron/folic acid	**√**	**√**	**√**	**√**	**√**	**√**	**√**
Stockouts in past 6 months							
Chloroquine							
Quinine			v	**√**	**√**	**√**	**√**
SP			**√**	**√**	**√**	**√**	**√**
Functional thermometer	**√**	**√**		**√**	**√**	v	**√**
Functional microscope	**√**	**√**	**√**		**√**	**√**	**√**
Onsite malaria smear	**√**	**√**	**√**		**√**	**√**	**√**

**√**= present. PW = pregnant woman; SP = sulfadoxine-pyrimethamine.

*Distributed at adjacent office of the district malaria officer but not specifically targeted to pregnant women.

There was considerable variability in the availability and distribution of bednets at the participating ANCs. In Jharkhand, bednets were only available in Gumla. An average of 2200 ITNs was distributed by the office of the district malaria officer that was housed in the same medical complex as the ANC at Civil Hospital in Gumla. Notably, bednets were not distributed specifically to pregnant women at the time of an antenatal visit and, as documented in the HCW observations, none of the HCWs recommended bednet use or specifically referred women to the adjacent malaria office for procurement of a bednet. In contrast, all facilities in Chhattisgarh had bednets available for distribution to pregnant women, with average monthly distribution ranging from 5 to 200. All of the nets distributed in Dongargarh and Keskal were insecticide-treated, only some were insecticide-treated in Rajnandgaon, and none were treated in Kondagaon. Retreatment kits were available at only 2 of the 7 facilities. Where available, bednets were distributed free of charge.

## Discussion

Malaria prevention and treatment during pregnancy is only one component of antenatal care and must compete with the other myriad demands on antenatal providers such as providing nutritional advice, screening for hypertensive complications of pregnancy, treating anemia and identifying obstetric complications. Nonetheless, this study highlights sporadic gaps in malaria diagnostic and treatment capabilities and an overall underutilization of malaria prevention strategies at the 7 participating antenatal clinics in eastern India.

### Case Management

In areas of both stable and unstable malaria transmission, timely diagnosis of malaria episodes is crucial to preventing the adverse sequelae of such infections during pregnancy. At the time that the surveys were conducted, the Directorate of Health Services in both states--Jharkhand and Chhattisgarh--recommended early diagnosis and treatment with effective antimalarials for pregnant women attending ANCs [18,19], guidelines that were consistent with those advised by the National Vector Borne Disease Control Program [16]. All participating facilities had onsite capability to perform malaria blood smears and, with the exception of one site whose microscope was broken, the equipment was functional and the relevant supplies well stocked. Rapid diagnostic tests were not used in any of the study ANCs. These might be a useful addition to the repertoire of diagnostic tools, particularly in facilities that lack the onsite capability to perform or interpret malaria blood smears [23].

Blood smears were not routinely ordered but more typically obtained in situations where fever or other malarial symptoms were divulged. Yet, queries about the presence of fever were made in only a minority of the ANC visits (36%), thus highlighting a potential way to improve case recognition. Asking each pregnant client about current or past fevers since the last visit and reflexively ordering blood smears for those who respond affirmatively would add minimal additional time to a routine visit and may be a more realistic strategy in areas of unstable malaria transmission when compared with routine collection of blood smears. Although our study did not ascertain the proportion of moderate to severe anemic women who were subsequently screened for malaria, such targeted blood smears would be an additional relevant consideration for improving case recognition among women with underlying partial immunity who might not mount a febrile response to the parasite. Such targeted blood smears, in the presence of a fever or moderate to severe anemia, should be considered by the government in future antenatal policy recommendations.

In addition to malaria diagnostic capacity, case management requires ready access to effective antimalarial drug regimens suitable for a pregnant population. Chloroquine, with its longstanding safety profile in pregnancy [24], was widely available at all of the antenatal facilities in relatively ample supply with only a single stockout at one facility. Nonetheless, chloroquine resistance has been reported in India, as elsewhere, particularly in areas of intense *P. falciparum *transmission such as the northeastern states [6,12]. Artemisinin combination therapy was introduced for nonpregnant individuals as early as 2006 by the government in areas showing chloroquine resistance [25]. Alternatives to chloroquine for malaria treatment during pregnancy are urgently needed yet we found that other antimalarials, including quinine, sulfadoxine-pyrimethamine and artemisinin derivatives, were much less widely available. Oral artesunate was available at only one site. By contrast, arte-ether for parenteral treatment of severe malaria was available at all seven study sites. Furthermore, little guidance is provided to HCWs caring for pregnant women regarding appropriate antimalarial treatment in the National Drug Policy on Malaria 2007 guidelines available at the time our study was conducted [16]. Certain antimalarials are prohibited from use during pregnancy--primaquine and parenteral artemesin derivatives. Yet, recommendations for which regimens to use for treatment of complicated or uncomplicated malaria during pregnancy are notably absent. To improve appropriate treatment of women with malaria in pregnancy, government agencies should outline treatment guidelines specific to pregnancy and target educational efforts to antenatal providers.

### Malaria Prevention Measures

A keystone of the Enhanced Malaria Control Program, active in both Jharkhand and Chattisgarh just prior to the initiation of our study, was the enhanced provision of ITNs and their regular retreatment. In addition to a policy of increasing ITN use, both state governments also had existing vector control strategies that primarily consisted of indoor residual spraying with either dichlorodiphenyltrichloroethane (DDT) or synthetic pyrethroids [18,19]. It was encouraging to note that a culture of bednet use by pregnant women existed in Jharkhand despite the fact that distribution occurred at none of the facilities during routine antenatal visits. We acknowledge that bednet use was self-reported and not verified by research staff. It is possible that women may have over-reported their use of bednets since they were aware of the study's focus upon malaria.

Availability of bednets at ANCs was higher in Chhattisgarh, but the majority of pregnant women did not regularly sleep under the nets. Except in Bastar district, there was an almost universal lack of insecticide treatment of the nets in both Jharkhand and Chhattisgarh. Approaches to bednet distribution, particularly ITNs, and improved community awareness of the importance of their use need to be addressed. Long-lasting ITNs may be preferable given the additional challenges of retreatment. The ANC visit is an ideal setting for distribution of ITNs and education in the importance of their use during pregnancy since the majority of pregnancy women attend at least one antenatal visit, 85% in Jharkhand [26] and 80% in Chhattisgarh [27].

Weekly chloroquine prophylaxis has generally fallen out of favor worldwide given several drawbacks: increased parasite resistance to the drug with consequent decreased efficacy and poor adherence to the prolonged regimen [28-30]. Chloroquine prophylaxis was rarely utilized for prevention of malaria in pregnancy at our study sites despite being recommended officially at the time the study was conducted [16]. This recommendation subsequently was dropped in the most recent Indian national drug policy guidelines on malaria, which were published in 2008 [31].

An alternative to weekly chemoprophylaxis widely adopted in Africa is IPTp, a strategy which delivers at least two curative doses of antimalarial treatment beyond the first trimester. Sulfadoxine-pyrimethamine is the most commonly administered drug for IPTp. The advantages of IPTp over weekly chemoprophylaxis include improved adherence and ease of delivery. Sulfadoxine-pyrimethamine was not widely available at the participating ANCs as it was not officially endorsed by the government for preventive use during pregnancy. The current national drug policy guidelines on malaria do not mention IPTp [31]. Given that the intensity of transmission is lower in Jharkhand [20] and Chhattisgarh [21,22] relative to many areas of sub-Saharan Africa, widespread implementation may unnecessarily expose pregnant women and their fetuses to the medications. However, there may be circumstances that warrant IPTp use, for example more intense transmission in remote tribal villages or during episodic outbreaks.

Participating HCWs and pregnant women were cognizant that study personnel were affiliated with a malaria research study which may have biased our results by overinflating the emphasis on malaria during ANC visits. Nevertheless, this study suggests that a disconnect remains between routine antenatal practices in India and known strategies to prevent and treat malaria in pregnancy. The use of ITNs in particular, which may be useful even in settings of unstable transmission, appears underutilized. Maintaining a sufficient supply of malaria diagnostic materials and antimalarials safe for use in pregnancy is crucial to effective case recognition and management. Gaps highlighted by this study combined with recent estimates of the prevalence of malaria in pregnancy specific to India should be used to revise governmental policy and target increased educational efforts among health care workers and pregnant women.

## Conclusion

This study identified a major disconnect between routine antenatal practices in India and known strategies to prevent and treat malaria in pregnancy. These gaps, combined with recent estimates of the prevalence of malaria in pregnant women in India, should be used to revise governmental policy and target increased educational efforts among health workers and pregnant women. Specifically, we recommend that HCWs routinely query pregnant women at each ANC visit about the presence of a fever and obtain a targeted blood smear for women with this history. In addition, there should be an increased focus at ANC visits upon distribution of insecticide-treated bednets along with improved education surrounding the benefits of utilization during pregnancy. Finally, national drug policy guidelines for the treatment of malaria should include specific recommendations for safe antimalarials for treatment during pregnancy and specify those situations, such as episodic outbreaks, where preventive therapy may be warranted.

## Abbreviations

The following abbreviations were used in the manuscript: ANC: antenatal clinic; HCW: health care worker; ITN: insecticide-treated bednet; IPTp: intermittent preventive therapy of pregnancy.

## Competing interests

Davidson Hamer has one competing interest to declare, specifically equity ownership in Inverness Medical Innovations, a company that produces a malaria rapid diagnostic test. All other authors declare that they have no competing interests.

## Authors' contributions

BJW, NS, LS, KYA, MD, VU, and DHH contributed to the conception and design of the study. AHH, MPS, JT, MH, CB, MIB, APD and NS all participated in study implementation and data collection. BJW, MPS, and WBM performed data analyses and with AHH, NS, LS, KYA, MIB, MD, VU, and DHH assisted with interpretation of the data. BJW, AHH, and DHH drafted the manuscript. All authors contributed to revisions of the manuscript and read and approved the final manuscript.

## Pre-publication history

The pre-publication history for this paper can be accessed here:

http://www.biomedcentral.com/1471-2458/10/557/prepub
